# Safety and Feasibility of Serial Lumbar Punctures: Long-term Results from the Parkinson’s Progression Markers Initiative

**DOI:** 10.1016/j.prdoa.2025.100385

**Published:** 2025-08-13

**Authors:** William Barbosa, Ruth B Schneider, Seung Ho Choi, Micah J Marshall, David-Erick Lafontant, Chelsea Caspell-Garcia, Christopher S Coffey, Jason Ross, Andrew Siderowf, Kenneth Marek, Tanya Simuni, on behalf of The Parkinson’s Progression Markers Initiative (PPMI)

**Affiliations:** a University of Rochester Medical Center, Rochester, NY, United States; b University of Iowa College of Public Health, Department of Biostatistics, Iowa City, IA, United States; c Northwestern University Feinberg School of Medicine, Chicago, IL, United States; d University of Pennsylvania Perelman School of Medicine, Philadelphia, PA, United States; e Institute of Neurodegenerative Disorders, New Haven, CT, United States

**Keywords:** Parkinson’s disease, α-synuclein, Cerebrospinal fluid, Lumbar puncture, Feasibility

## Abstract

**Background:**

Cerebrospinal fluid (CSF) serves an essential role in biomarker research. New Parkinson’s disease (PD) classifications incorporate CSF α-synuclein status into trial design. This study evaluated the safety and feasibility of serial CSF collection in participants enrolled in the Parkinson’s Progression Markers Initiative (PPMI).

**Methods:**

PPMI participants were evaluated over 13-years with lumbar punctures (LPs) occurring annually from baseline through year five and biennially thereafter. Adverse events and compliance, defined as percentage of LPs with CSF collection, were assessed at baseline and upon follow up. Logistic regression and generalized linear mixed effects models were used to calculate odds ratios and 95% confidence intervals for predictors of baseline and longitudinal LP success.

**Results:**

3479 participants (PD: n = 1412, prodromal: n = 1768, healthy control: n = 299) were analyzed. 3360 attempted at least one LP with 29.5 % experiencing an adverse event (1.3 % severe). Baseline compliance was 90 %. From baseline to year five, percent change in compliance decreased by 39.4 % in the PD cohort, 41.4 % in the prodromal cohort, and 27.8 % in the healthy control cohort. Predictive variables of baseline LP success included fewer years since diagnosis (PD: OR 0.82, 0.76–0.89), lower BMI (prodromal: OR 0.92, 0.89–0.94), and site location U.S. vs. non-U.S. (PD: OR 1.5, 1.03–2.18, healthy control: OR 3.6, 1.22–10.64). Baseline LP success was the best predictor of longitudinal success (OR 7.82, 5.74–10.65).

**Conclusions:**

Lumbar punctures were safe in PD research participants over a 13-year period. Compliance was high over the first three years, but further investigation is warranted to improve long term success.

## Highlights

• Clinical studies are incorporating cerebrospinal fluid biomarker metrics into trial design.
• Longitudinal analysis of lumbar puncture safety and feasibility is limited.
• In a large cohort study, serial lumbar punctures were safe over a 13-year period.
• Compliance is high over the first three years but further investigation is warranted to improve long term success.

## 1 Introduction

The collection of cerebrospinal fluid (CSF) serves an essential role in the investigation of biomarkers of neurodegenerative disease [1,2]. Pathologic changes in the brain are reliably expressed in the CSF, often before clinical symptoms become manifest making this an ideal source for biological disease characterization [3]. Interventional studies are increasingly incorporating CSF biomarker metrics as part of their trial design and it is anticipated that CSF collection will serve an important role in recruiting populations of interest in future disease prevention trials [4]. Historically Parkinson’s disease (PD) has been a clinical diagnosis, but the recent development of an α-synuclein seed amplification assay (SAA) marks a significant breakthrough [5].

Newly proposed biological definitions and research classifications of PD include CSF α-synuclein SAA status as a key biological anchor [6,7]. The collection of CSF via lumbar punctures (LPs) is expected to become integral to PD clinical trials and potentially in clinical practice as we continue to characterize and expand upon our understanding of biomarker assays. Currently, the CSF-SAA is being implemented to enrich research trial enrollment and identify participants who may benefit from targeted disease modifying intervention [8]. Longitudinal CSF collection can be utilized to assess target engagement, measure therapeutic efficacy over time, and identify co-pathological biomarkers of disease progression [9].

As progress is made towards PD prevention trials, it remains imperative to understand the safety and feasibility of longitudinal LPs among individuals at risk for disease progression. While previous reports have established the safety and feasibility of LPs at baseline in the Parkinson’s Progression Markers Initiative (PPMI)[10], analysis of prodromal cohorts and longitudinal investigation across disease stages is needed. Here we evaluate the safety and feasibility of serial CSF collection in participants with PD, without PD, and with prodromal disease status enrolled in PPMI. In addition, we examine demographic, clinical, and LP-related characteristics to identify potential predictors of longitudinal LP success to help inform future clinical trial design.

## 2 Methods

PPMI is an ongoing observational, multi-center, international study designed to identify clinical, genetic, imaging, and biological markers of PD progression to support advances in treatment. The PPMI study design, methods, and eligibility criteria have been reported previously and are available at https://www.ppmi-info.org/study-design. At each study site informed consent was provided by all participants as in accordance with good clinical practices and research ethics board approval. Participants undergo LPs annually from baseline through year five and biennially thereafter with CSF collection performed predominantly using 24-gauge needles by trained study site investigators. Fluoroscopy utilization was determined as per site investigator assessment.

For the purposes of this study, we analyzed data from PPMI participants enrolled in the PD (sporadic, leucine rich repeat kinase 2 (LRRK2), glucocerebrosidase (GBA), rare genetic), prodromal (REM sleep behavior disorder (RBD), hyposmia, LRRK2, GBA, rare genetic), and healthy control cohorts. Participants with certain genetic variants were allowed to enroll without an LP in order to maximize recruitment of participants with rare genetic variants. LPs were conducted over a 13-year period across all scheduled LP visits. Baseline demographic variables were summarized and stratified by subgroup to include age, sex, BMI, race, and ethnicity. Clinical variables included the number of years since diagnosis, Movement Disorders Society Unified Parkinson’s Disease Rating Scale I-III scores, Hoehn & Yahr, and Montreal Cognitive Assessment. CSF collection metrics examined included total fluid volume, incidence of traumatic LPs, and fluoroscopy utilization.

Adverse events (AEs) related to LPs were examined for all participants who attempted at least one LP procedure. The occurrence of AEs was assessed at the time of the study visit and again within one week following the procedure. AEs were summarized by event type, site-reported severity, and cohort according to established guidelines. Mild AEs were classified as typically transient events that generally did not interfere with normal activities. Moderate AEs caused sufficient discomfort to interfere with normal activities. Severe AEs were events that incapacitated participants thus preventing them from performing normal activities. Compliance, defined as CSF collection status of “Collected” or “Partial Collection” in eligible participants, was examined at baseline and upon longitudinal follow up. Reasons for CSF collection not completed were further evaluated by cohort and study visit.

To assess the association between baseline and longitudinal success, odds ratios, associated 95 % Wald confidence intervals and p-values comparing the odds of at least one compliant post-baseline visit given baseline compliance vs. non-compliance were computed. To identify specific baseline predictors of LP success, two sets of models were run for each cohort: logistic regression models to predict baseline success, and generalized linear mixed effects models with binomial distribution and logit link to predict longitudinal LP success. Candidate predictors included age, sex, years since diagnosis (PD cohort only), BMI, race, ethnicity, site (US vs. non-US), and a two-way interaction between sex and BMI. Interactions with time were also considered in the longitudinal models. Utilizing an initial screening step that included covariates that were significant at α = 0.10 in univariate/screening models, a backward selection procedure was employed to sequentially remove variables until all remaining variables (or their interaction with time) were significant at α = 0.05. Longitudinal models with LP method (fluoroscopy vs. non-fluoroscopy) and occurrence of LP-related AE within 7 days of baseline LP as predictors were run separately in the subset of participants who had a successful baseline LP. Odds ratios, associated 95 % confidence intervals, and p-values are reported in the final models. Longitudinal models were estimated using Laplace estimation with unstructured covariance structure and inclusion of random intercept and slope terms. Statistical analyses were performed using SAS v9.4 (SAS Institute Inc., Cary, NC, USA; sas.com; RRID:SCR_008567). Data was downloaded on June 17, 2024.

## 3 Results

3479 PPMI participants enrolled in the PD (n = 1412), prodromal (n = 1768), and healthy control (n = 299) cohorts were analyzed. Baseline age, sex, race, and BMI variables were comparable across cohorts (median age of 65 years, 55 % male, 94 % White, and median BMI 26.6). For all study participants baseline LP compliance with either collected or partially collected CSF was 90 %, of which 13 % were fluoroscopy guided and 10 % were traumatic. Lower baseline compliance percentages of 78 % and 66 % were observed in the GBA PD and rare genetic variant PD subgroups respectively. Further baseline demographics and clinical characteristics of study participants stratified by subgroup are summarized in Table 1.

**Table 1:** Baseline demographics, disease characteristics, and lumbar puncture status.

		**Parkinson's Disease**				**Prodromal**					**Healthy Control**^c^
**Variable**	**All Participants(N = 3479)**	**Sporadic(N = 1062)**	**LRRK2(N = 175)**	**GBA(N = 110)**	**Rare Genetic**^a^**(N = 65)**	**RBD(N = 399)**	**Hyposmia(N = 932)**	**LRRK2(N = 212)**	**GBA(N = 189)**	**Rare Genetic**^b^**(N = 36)**	
**Age at baseline, years**, median (IQR)	65.3 (60.4–70.4)	64.2 (57.2–70.1)	65.4 (58.0–70.0)	62.7 (54.8–69.6)	54.0 (47.8–58.2)	68.5 (64.2–72.2)	67.2 (63.7–71.4)	61.4 (55.9–66.5)	61.7 (57.3–66.8)	60.9 (50.7–69.0)	63.7 (56.3–69.9)
**Male sex**, n (%)	1917 (55 %)	695 (65 %)	85 (49 %)	61 (55 %)	30 (46 %)	310 (78 %)	373 (40 %)	90 (42 %)	74 (39 %)	14 (39 %)	185 (62 %)
**Body mass index**, median (IQR)	26.6 (24.0–30.1)	26.2 (23.9–29.4)	26.2 (23.4–29.8)	26.3 (23.8–29.4)	25.8 (23.4–30.7)	26.8 (24.6–30.0)	27.2 (24.3–31.0)	26.7 (23.5–30.7)	26.5 (24.2–30.3)	26.3 (24.3–28.9)	26.3 (23.7–29.7)
**Race**, n (%)											
White	3244 (94 %)	983 (93 %)	164 (94 %)	107 (97 %)	65 (100 %)	372 (94 %)	870 (94 %)	200 (97 %)	185 (98 %)	35 (97 %)	263 (88 %)
Black or African American	58 (2 %)	21 (2 %)	0	1 (1 %)	0	9 (2 %)	8 (1 %)	0	0	0	19 (6 %)
Asian	33 (1 %)	16 (2 %)	1 (1 %)	0	0	8 (2 %)	1 (<1%)	0	0	0	7 (2 %)
American Indian or Alaska Native	3 (<1%)	1 (<1%)	0	0	0	1 (<1%)	0	0	0	0	1 (<1%)
Other	27 (1 %)	12 (1 %)	2 (1 %)	0	0	1 (<1%)	5 (1 %)	2 (1 %)	2 (1 %)	0	3 (1 %)
Multiracial	87 (3 %)	22 (2 %)	8 (5 %)	2 (2 %)	0	4 (1 %)	37 (4 %)	5 (2 %)	2 (1 %)	1 (3 %)	6 (2 %)
**Hispanic or Latino ethnicity**, n (%)	168 (5 %)	46 (4 %)	30 (17 %)	1 (1 %)	4 (6 %)	37 (10 %)	21 (2 %)	18 (8 %)	5 (3 %)	2 (6 %)	4 (1 %)
**Years since diagnosis**, median (IQR)	0.7 (0.3–1.5)	0.5 (0.3–1.0)	2.4 (1.1–4.5)	2.2 (0.7–4.6)	2.8 (0.4–5.8)	–	–	–	–	–	–
**MDS-UPDRS Part I**, median (IQR)	5.0 (3.0–9.0)	5.0 (3.0–9.0)	7.0 (3.0–11.0)	7.0 (3.0–11.0)	7.0 (3.0–11.0)	7.0 (4.0–10.0)	6.0 (3.0–9.0)	4.0 (2.0–6.0)	4.0 (2.0–8.0)	4.5 (2.0–8.0)	3.0 (1.0–5.0)
**MDS-UPDRS Part II**, median (IQR)	2.0 (0.0–6.0)	5.0 (3.0–9.0)	6.0 (3.0–9.0)	6.0 (3.0–12.0)	6.0 (3.0–12.0)	1.0 (0.0–4.0)	1.0 (0.0–3.0)	0.0 (0.0–1.0)	0.0 (0.0–1.0)	0.5 (0.0–2.0)	0.0 (0.0–0.0)
**MDS-UPDRS Part III (OFF)**, median (IQR)	7.0 (2.0–19.0)	21.0 (15.0–29.0)	19.0 (14.0–27.0)	23.0 (17.0–31.0)	18.0 (13.0–24.0)	3.0 (1.0–7.0)	3.0 (1.0–7.0)	2.0 (0.0–4.0)	1.0 (0.0–4.0)	0.0 (0.0–2.0)	0.0 (0.0–2.0)
Missing	125	5	41	23	12	8	24	3	2	3	4

**Hoehn & Yahr (OFF)**, n (%)											
0	1868 (55 %)	2 (<1%)	1 (1 %)	2 (2 %)	0	374 (94 %)	788 (85 %)	196 (94 %)	181 (96 %)	33 (97 %)	291 (98 %)
1	539 (16 %)	368 (35 %)	33 (25 %)	28 (32 %)	18 (34 %)	8 (2 %)	62 (7 %)	12 (6 %)	4 (2 %)	0	6 (2 %)
2	946 (28 %)	687 (65 %)	90 (67 %)	51 (59 %)	30 (57 %)	14 (4 %)	69 (7 %)	1 (<1%)	3 (2 %)	1 (3 %)	0
3	28 (1 %)	4 (<1%)	10 (7 %)	6 (7 %)	5 (9 %)	0	3 (<1%)	0	0	0	0
4	2 (<1%)	0	0	0	0	0	2 (<1%)	0	0	0	0
Missing	96	1	41	23	12	3	8	3	1	2	2
**MoCA**, median (IQR)	27.0 (25.0–29.0)	27.0 (26.0–29.0)	27.0 (24.0–28.0)	27.0 (25.0–28.0)	27.0 (25.0–29.0)	27.0 (25.0–28.0)	27.0 (25.0–28.0)	27.0 (25.0–29.0)	27.0 (26.0–28.0)	28.0 (25.5–29.0)	28.0 (27.0–29.0)

**CSF collected**, n (%)											
Collected	2971 (85 %)	941 (89 %)	155 (89 %)	75 (68 %)	40 (62 %)	347 (87 %)	784 (84 %)	176 (83 %)	157 (83 %)	27 (75 %)	269 (90 %)
Partial collection	160 (5 %)	49 (5 %)	5 (3 %)	11 (10 %)	3 (5 %)	15 (4 %)	41 (4 %)	11 (5 %)	8 (4 %)	1 (3 %)	16 (5 %)
Attempted, no collection	215 (6 %)	39 (4 %)	7 (4 %)	8 (7 %)	3 (5 %)	21 (5 %)	89 (10 %)	19 (9 %)	17 (9 %)	0	12 (4 %)
Not done	88 (3 %)	10 (1 %)	4 (2 %)	13 (12 %)	17 (26 %)	10 (3 %)	13 (1 %)	6 (3 %)	6 (3 %)	7 (19 %)	2 (1 %)
Missing, LP expected	45 (1 %)	23 (2 %)	4 (2 %)	3 (3 %)	2 (3 %)	6 (2 %)	5 (1 %)	0	1 (1 %)	1 (3 %)	0
Fluoroscopy*, n (%)	400 (13 %)	137 (14 %)	9 (6 %)	3 (3 %)	1 (2 %)	63 (17 %)	140 (17 %)	9 (5 %)	7 (4 %)	2 (7 %)	29 (10 %)
CSF volume*, median (IQR)	16.0 (14.0–19.0)	16.0 (14.0–19.0)	15.0 (15.0–19.0)	16.5 (13.0–20.0)	14.0 (11.0–17.0)	15.0 (13.0–19.0)	16.0 (14.0–19.0)	17.0 (14.0–20.0)	18.0 (16.0–21.0)	15.0 (12.0–18.0)	15.0 (14.0–18.0)
Bloody tap*, n (%)	327 (10 %)	107 (11 %)	18 (11 %)	3 (3 %)	6 (14 %)	44 (12 %)	86 (10 %)	16 (9 %)	11 (7 %)	1 (4 %)	35 (12 %)

Report generated on data submitted as of: 17JUN2024.

MDS-UPDRS = Movement Disorder Society-Unified Parkinson's Disease Rating Scale; MoCA = Montreal Cognitive Assessment. Percentages are not reported for groups with less than 20 participants.

*Missing data* (for variables where < 5 % missing in all subgroups): Age *n* = 3; Sex *n* = 3; Body mass index *n* = 43; Race *n* = 27; Hispanic or Latino ethnicity *n* = 44; Years since diagnosis *n* = 2; MDS-UPDRS Part I *n* = 30; MDS-UPDRS Part II *n* = 10; MoCA *n* = 37; CSF volume *n* = 6; Bloody tap *n* = 2.

* Only includes participants for whom CSF was at least partially collected.

a Includes, 31 SNCA, 24 PRKN, 9 LRRK2 + GBA, 1 PINK1.

b Includes 20 LRRK2 + GBA, 11 SNCA, 2 PRKN, 1 LRRK2 + PINK1, 1 LRRK2 + VPS35, 1 PARK7.

c Includes 4 participants found to have genetic mutations: 2 GBA, 1 PINK1, 1 PRKN.

Among all participants who attempted at least one LP (n = 3360), 29.5 % experienced an adverse event **(**Table 2**)**. The most common adverse events were headache (16.0 %) and back/injection site pain (14.1 %). The healthy control cohort exhibited a higher proportion of adverse events (44.6 %) when compared to PD (31.4 %) and prodromal (25.3 %) cohorts. Examination of adverse event severity of all participants revealed that 22.9 % (n = 769) experienced a mild event, 8.9 % (n = 299) experienced a moderate event, and 1.3 % (n = 43) experienced a severe event.

**Table 2:** Lumbar puncture-related adverse events by type, severity and cohort.

	**All Participants (N = 3360)**				**Parkinson's Disease (N = 1341)**				**Prodromal (N = 1721)**				**Healthy Control (N = 298)**			
**Adverse Event**	**# of Ps.**	**% of Ps.**	**# of Events**	**Rate**	**# of Ps.**	**% of Ps.**	**# of Events**	**Rate**	**# of Ps.**	**% of Ps.**	**# of Events**	**Rate**	**# of Ps.**	**% of Ps.**	**# of Events**	**Rate**
**Total**	**990**	**29.5 %**	**1669**	**0.021**	**421**	**31.4 %**	**707**	**0.018**	**436**	**25.3 %**	**719**	**0.027**	**133**	**44.6 %**	**243**	**0.017**
*Mild*	*769*	*22.9 %*	*1234*	*0.015*	*323*	*24.1 %*	*500*	*0.013*	*350*	*20.3 %*	*571*	*0.021*	*96*	*32.2 %*	*163*	*0.012*
*Moderate*	*299*	*8.9 %*	*384*	*0.005*	*134*	*10.0 %*	*182*	*0.005*	*116*	*6.7 %*	*136*	*0.005*	*49*	*16.4 %*	*66*	*0.005*
*Severe*	*43*	*1.3 %*	*51*	*0.0006*	*22*	*1.6 %*	*25*	*0.0006*	*11*	*0.6 %*	*12*	*0.0004*	*10*	*3.4 %*	*14*	*0.001*
Headache	538	16.0 %	666	0.008	242	18.0 %	321	0.008	226	13.1 %	255	0.009	70	23.5 %	90	0.006
*Mild*	*367*	*10.9 %*	*444*	*0.006*	*167*	*12.5 %*	*207*	*0.005*	*159*	*9.2 %*	*181*	*0.007*	*41*	*13.8 %*	*56*	*0.004*
*Moderate*	*175*	*5.2 %*	*190*	*0.002*	*84*	*6.3 %*	*98*	*0.002*	*64*	*3.7 %*	*65*	*0.002*	*27*	*9.1 %*	*27*	*0.002*
*Severe*	*31*	*0.9 %*	*32*	*0.0004*	*15*	*1.1 %*	*16*	*0.0004*	*9*	*0.5 %*	*9*	*0.0003*	*7*	*2.3 %*	*7*	*0.0005*
Back or injection site pain	475	14.1 %	667	0.008	176	13.1 %	236	0.006	230	13.4 %	319	0.012	69	23.2 %	112	0.008
*Mild*	*411*	*12.2 %*	*554*	*0.007*	*153*	*11.4 %*	*196*	*0.005*	*200*	*11.6 %*	*278*	*0.010*	*58*	*19.5 %*	*80*	*0.006*
*Moderate*	*89*	*2.6 %*	*106*	*0.001*	*32*	*2.4 %*	*38*	*0.0010*	*37*	*2.1 %*	*40*	*0.001*	*20*	*6.7 %*	*28*	*0.002*
*Severe*	*7*	*0.2 %*	*7*	*0.0001*	*2*	*0.1 %*	*2*	*0.0001*	*1*	*0.1 %*	*1*	*0<.0001*	*4*	*1.3 %*	*4*	*0.0003*
Other	260	7.7 %	336	0.004	114	8.5 %	150	0.004	111	6.4 %	145	0.005	35	11.7 %	41	0.003
*Mild*	*196*	*5.8 %*	*236*	*0.003*	*80*	*6.0 %*	*97*	*0.002*	*92*	*5.3 %*	*112*	*0.004*	*24*	*8.1 %*	*27*	*0.002*
*Moderate*	*69*	*2.1 %*	*88*	*0.001*	*35*	*2.6 %*	*46*	*0.001*	*24*	*1.4 %*	*31*	*0.001*	*10*	*3.4 %*	*11*	*0.0008*
*Severe*	*10*	*0.3 %*	*12*	*0.0001*	*6*	*0.4 %*	*7*	*0.0002*	*2*	*0.1 %*	*2*	*0.0001*	*2*	*0.7 %*	*3*	*0.0002*

Report generated on data submitted as of: 17JUN2024.

Column definitions:

# of Ps.: Number of participants who have ever had AE.

% of Ps.: Percentage of participants who have ever had AE.

# of Events: Total number of AEs; includes multiple AEs of same type per participant.

Rate: Number of AEs per 30 days.

Longitudinal examination across cohorts revealed compliance was 90 % at baseline, 76.3 % at year one, 67.8 % at year three, 56.2 % at year five, and 35.7 % at year nine **(**Supplemental Table 1**)**. From baseline to year five, percent change in compliance decreased by 39.4 % in the PD cohort, 41.4 % in the prodromal cohort, and 27.8 % in the healthy control cohort. The greatest drop off in compliance rates occurred between the baseline visit and year one, and between years five and seven **(**Supplemental Fig. 1**)**.

Odds ratios and 95 % confidence intervals for covariant predictors of baseline LP success were calculated with statistically significant results shown in Supplemental Table 2. Baseline variables of significance included years since diagnosis in the PD cohort (OR 0.82, 0.76–0.89), BMI in the prodromal cohort (OR 0.92, 0.89–0.94), and site location (U.S. vs. non-U.S.) for both PD (OR 1.50, 1.03–2.18) and healthy control (OR 3.60, 1.22–10.64) cohorts.

Baseline LP success was the most significant predictor of longitudinal LP success (OR 7.82, 5.74–10.65) (Supplemental Table 3). Across all cohorts, time from baseline predicted lower likelihood for longitudinal LP success with odds ratios of 0.46 (0.43–0.50), 0.66 (0.58–0.76), and 0.51 (0.45–0.59) in PD, prodromal, and healthy control cohorts respectively. For participants with a successful baseline LP, the occurrence of an LP-related AE within 7 days of baseline predicted lower likelihood of longitudinal LP success in the PD (OR 0.25, 0.11 – 0.56) and prodromal (OR 0.28, 0.15–0.52) cohorts (Supplemental Table 4). Amongst those who did not undergo a successful LP, participant refusal was the most cited reason for which CSF was not collected across all cohorts (Supplemental Table 5).

## 4 Discussion

In this large study of longitudinal CSF collection, serial lumbar punctures were safe across PD, prodromal PD, and healthy control research participants over a 13-year period. Most adverse events were of mild severity and only approximately 1 % of events were rated as severe. The most common adverse events were headache and back/site injection pain, which is in accordance with other multi-center baseline LP studies [11].

Baseline compliance for all participants was high with lower rates appreciated in the genetic cohort subgroups. This is likely due to their less stringent requirements for enrollment in PPMI as certain rare genetic cohort participants were allowed to be recruited despite LP refusal in attempt to maximize feasibility of study enrollment. Favorable predictors of baseline LP success were shorter interval time from diagnosis, having a lower BMI, and study site location in the United States. While prior studies have established a correlation between higher BMI and unsuccessful LP outcomes [12], the effects of time from diagnosis and study site location are not yet fully understood. Presumably greater time from diagnosis may result in additional comorbidities that could influence LP success. Additionally, there may be unrecognized study site differences in LP technique or informed consent procedures that could also impact success rates, but this requires further examination.

Over the first three years longitudinal compliance was high but exhibited steady attrition across cohorts over time. Participant refusal remained the most cited reason for unsuccessful CSF collection, but other potential contributors to consider include loss of motivation, escalating symptom burden, lack of time/resources, or prior negative LP experience. Interestingly, healthy controls demonstrated the highest baseline and longitudinal compliance despite having a greater percentage of adverse events. While it is unclear why healthy controls exhibited higher rates of adverse events, these results further highlight the importance of maximizing baseline LP success as this was the best predictor of long-term compliance. To ensure high compliance rates, PPMI committed substantial resources to training sites and standardization of procedures. Additional implemented strategies included providing standardized information packages and videos, and establishing a centralized LP education core to offer consistent information at the participant friendly level.

While notable strengths of this study include the large sample size, extended duration of follow up, and comprehensive evaluation of factors associated with successful LPs, there are also limitations worth acknowledging. First there was lack of racial and ethnic diversity among the enrolled participants. Additionally, the rate of adverse events may be influenced by the number of needle punctures per LP attempt but data on this metric was not collected. While rating of adverse event severity was graded according to commonly accepted research standards, some subjectivity in assessment and report of symptoms remains. Similarly, the determination as to which participants were selected to undergo fluoroscopy occurred at the site level and was not standardized. Examination of site level differences was restricted to whether or not CSF collection took place in the United States. Although participant refusal was the most cited reason for unsuccessful CSF collection, data were not collected to understand the reason for refusal. This allows for limited opportunity to understand the rationale for declining the procedure. This limitation is compounded by the significant percentage of responses for which no reason was provided for why CSF was not collected. Lastly, within the prodromal and genetic subgroup cohorts there is shorter duration of follow up as recruitment of these groups was initiated a few years following study launch.

In conclusion, although the safety profile for LPs in PPMI is reassuring and compliance for serial LPs is high over the first three years, further investigation is warranted to explore methods to improve long-term retention. The drop in LP compliance over time may present challenges in the conduct of future disease-modifying trials. Efforts to better understand the rationale for participant refusal and examination of site level differences may highlight how best to minimize study attrition moving forward. Clarifying identified participant uncertainties or misconceptions through educational materials, readdressing best practices for the process of informed consent, further standardizing LP training protocols, and guidelines for fluoroscopy utilization may prove to be effective methods to ensure longitudinal LP success.

## CRediT authorship contribution statement

**William Barbosa:** Writing – original draft, Methodology, Data curation, Conceptualization. **Ruth B Schneider:** Writing – review & editing, Methodology, Data curation, Conceptualization. **Seung Ho Choi:** Writing – review & editing, Formal analysis, Data curation. **Micah J Marshall:** Formal analysis, Data curation. **David-Erick Lafontant:** Formal analysis, Data curation. **Chelsea Caspell-Garcia:** Formal analysis, Data curation. **Christopher S Coffey:** Supervision, Formal analysis, Data curation. **Jason Ross:** Writing – review & editing. **Andrew Siderowf:** Writing – review & editing, Methodology, Conceptualization. **Kenneth Marek:** Methodology, Conceptualization. **Tanya Simuni:** Writing – review & editing, Supervision, Methodology, Data curation, Conceptualization.

## Declaration of competing interest

The authors declare the following financial interests/personal relationships which may be considered as potential competing interests: WB declares research funding from the Michael J. Fox Foundation for Parkinson's Research.

RBS declares research funding from the National Institutes of Health, Michael J. Fox Foundation for Parkinson’s Research, Parkinson’s Foundation, Bial, Biohaven Pharmaceuticals, and Acadia Pharmaceuticals. She has participated on a Sun Pharma Advisory Board and received funding from Clintrex Research Corporation for participation on a DSMB.

SHC declares research funding to his institution from The Michael J. Fox Foundation and NIH/NINDS.

MM declares research funding to his institution from The Michael J. Fox Foundation.

DEL declares research funding to his institution from The Michel J. Fox Foundation.

CCG declares research funding to her institution from The Michael J. Fox Foundation.

CSC declares grants from The Michael J. Fox Foundation and NIH/NINDS.

## Data Availability

Data used in the preparation of this article were obtained on June 17, 2024 from the Parkinson’s Progression Markers Initiative (PPMI) database (https://www.ppmi-info.org/access-data-specimens/download-data), RRID:SCR_006431. For up-to-date information on the study, visit https://www.ppmi-info.org. This analysis was conducted by the PPMI Statistics Core and used actual dates of activity for participants, a restricted data element not available to public users of PPMI data. Protocol information for The Parkinson’s Progression Markers Initiative (PPMI) Clinical − Establishing a Deeply Phenotyped PD Cohort AM 3.2. can be found on protocols.io or by following this link: https://dx.https://doi.org/10.17504/protocols.io.n92ldmw6ol5b/v2. Statistical analysis codes used to perform the analyses in this article are shared on Zenodo (https://doi.org/10.5281/zenodo.15079775).
