# Supplementary material for: Safety and Feasibility of Serial Lumbar Punctures: Long-term Results from the Parkinson’s Progression Markers Initiative
Source: Clin Park Relat Disord. 2025 Aug 13;13:100385. doi: 10.1016/j.prdoa.2025.100385 (PMC12391599; doi:10.1016/j.prdoa.2025.100385)
Supplement: Supplementary Data 2 [file mmc2.docx]

**Supplemental Table 1. Longitudinal CSF compliance by group**

|  | | **Parkinson's Disease** | | | | **Prodromal** | | | | | **Healthy Control^3^** |
| --- | --- | --- | --- | --- | --- | --- | --- | --- | --- | --- | --- |
| **Visit** | **All Participants** | **Sporadic** | **LRRK2** | **GBA** | **Rare Genetic^1^** | **RBD** | **Hyposmia** | **LRRK2** | **GBA** | **Rare Genetic^2^** |  |
| Baseline | 3131/3479 (90.0%) | 990/1062 (93.2%) | 160/175 (91.4%) | 86/110 (78.2%) | 43/65 (66.2%) | 362/399 (90.7%) | 825/932 (88.5%) | 187/212 (88.2%) | 165/189 (87.3%) | 28/36 (77.8%) | 285/299 (95.3%) |
| Year 1 | 1611/2111 (76.3%) | 560/724 (77.3%) | 110/154 (71.4%) | 49/96 (51.0%) | 24/51 (47.1%) | 146/187 (78.1%) | 211/267 (79.0%) | 157/191 (82.2%) | 135/170 (79.4%) | 16/27 (59.3%) | 203/244 (83.2%) |
| Year 2 | 992/1390 (71.4%) | 375/490 (76.5%) | 86/133 (64.7%) | 44/78 (56.4%) | 18/40 (45.0%) | 49/71 (69.0%) | 39/54 (72.2%) | 115/163 (70.6%) | 91/129 (70.5%) | 14/23 (60.9%) | 161/209 (77.0%) |
| Year 3 | 729/1075 (67.8%) | 272/359 (75.8%) | 72/118 (61.0%) | 25/58 (43.1%) | 13/35 (37.1%) | 20/34 (58.8%) | 13/20 (65.0%) | 92/144 (63.9%) | 80/112 (71.4%) | 11/25 (44.0%) | 131/170 (77.1%) |
| Year 4 | 615/1006 (61.1%) | 215/317 (67.8%) | 61/113 (54.0%) | 31/56 (55.4%) | 12/31 (38.7%) | 14/34 (41.2%) | 12/20 (60.0%) | 86/139 (61.9%) | 67/116 (57.8%) | 9/18 (NA) | 108/162 (66.7%) |
| Year 5 | 534/951 (56.2%) | 185/299 (61.9%) | 48/96 (50.0%) | 23/61 (37.7%) | 9/27 (33.3%) | 7/20 (35.0%) | 6/15 (NA) | 77/133 (57.9%) | 68/130 (52.3%) | 5/16 (NA) | 106/154 (68.8%) |
| Year 7 | 239/576 (41.5%) | 83/225 (36.9%) | 19/64 (29.7%) | 9/20 (45.0%) | 2/12 (NA) | 4/12 (NA) | 4/11 (NA) | 33/65 (50.8%) | 30/48 (62.5%) | 3/12 (NA) | 52/107 (48.6%) |
| Year 9 | 95/266 (35.7%) | 43/124 (34.7%) | 8/40 (20.0%) | 1/3 (NA) | 3/7 (NA) | 2/10 (NA) | 5/12 (NA) | 11/17 (NA) | 0 | 1/2 (NA) | 21/51 (41.2%) |
| Year 11 | 77/245 (31.4%) | 38/143 (26.6%) | 1/4 (NA) | 3/4 (NA) | 3/4 (NA) | 0 | 0 | 0 | 0 | 0 | 32/90 (35.6%) |
| Year 13 | 7/39 (17.9%) | 2/15 (NA) | 1/3 (NA) | 0/1 (NA) | 0 | 0 | 0 | 0 | 0 | 0 | 4/20 (20.0%) |

Report generated on data submitted as of: 17JUN2024.

Compliance is defined as a CSF collection status of "Collected" or "Partial Collection". Percentages are not reported for groups with less than 20 participants.

^1^ Includes, 31 SNCA, 24 PRKN, 9 LRRK2 + GBA, 1 PINK1.

^2^ Includes 20 LRRK2 + GBA, 11 SNCA, 2 PRKN, 1 LRRK2 + PINK1, 1 LRRK2 + VPS35, 1 PARK7.

^3^ Includes 4 participants found to have genetic mutations: 2 GBA, 1 PINK1, 1 PRKN.

**Supplemental Table 2. Predictors of baseline lumbar puncture success**

| **Variable** | **OR (95% CI)** | ***p*** |
| --- | --- | --- |
| **Parkinson's Disease** | | |
| Years since diagnosis (1-year increase) | 0.82 (0.76, 0.89) | <.0001 |
| Site (US vs. non-US) | 1.50 (1.03, 2.18) | 0.0326 |
| **Prodromal** | | |
| BMI (1-unit increase) | 0.92 (0.89, 0.94) | <.0001 |
| **Healthy Control** | | |
| Site (US vs. non-US) | 3.60 (1.22, 10.64) | 0.0207 |

Report generated on data submitted as of: 17JUN2024.

The odds ratio (OR) and confidence interval (CI) estimates are obtained from logistic regression models using baseline LP success as the outcome.

**Supplementary Table 3. Association between baseline and longitudinal lumbar puncture success**

|  | **Longitudinal LP success among participants with:** | |  | |
| --- | --- | --- | --- | --- |
| **Cohort** | **Baseline success** | **Baseline non-success** | **OR (95% CI)** | ***p*** |
| Parkinson's Disease | 796/963 (82.7%) | 18/94 (19.1%) | 20.12 (11.73, 34.54) | <.0001 |
| Prodromal | 658/778 (84.6%) | 58/93 (62.4%) | 3.31 (2.08, 5.25) | <.0001 |
| Healthy Control | 217/240 (90.4%) | 4/9 (NA) | 11.79 (2.96, 47.03) | 0.0005 |
| Overall | 1671/1981 (84.4%) | 80/196 (40.8%) | 7.82 (5.74, 10.65) | <.0001 |

Report generated on data submitted as of: 17JUN2024.

Percentages are not reported for groups with less than 20 participants.

**Supplemental Table 4. Predictors of longitudinal lumbar puncture success**

| **Variable** | **OR (95% CI)** | ***p*** |
| --- | --- | --- |
| **Parkinson's Disease** | | |
| Time (1-year increase) | 0.46 (0.43, 0.50) | <.0001 |
| Years since diagnosis (1-year increase) | 0.74 (0.66, 0.82) | <.0001 |
| Site (US vs. non-US) | 3.15 (2.08, 4.77) | <.0001 |
| **For participants with successful baseline LP only** (separate model) |  |  |
| Occurrence of LP-related AE within 7 days of baseline LP | 0.25 (0.11, 0.56) | 0.0008 |
| Baseline LP-related AE x time interaction | 1.29 (1.10, 1.51) | 0.0018 |
| **Prodromal** | | |
| Time (1-year increase)* | 0.66 (0.58, 0.76) | <.0001 |
| Difference for a 1-year increase in age | 0.99 (0.97, 1.00) | 0.0397 |
| Difference for US vs. non-US site | 1.45 (1.12, 1.88) | 0.0044 |
| Age (1-year increase) | 1.00 (0.98, 1.03) | 0.7275 |
| BMI (1-unit increase) | 0.94 (0.91, 0.97) | 0.0003 |
| Site (US vs. non-US) | 1.61 (1.02, 2.54) | 0.0410 |
| **For participants with successful baseline LP only** (separate models) |  |  |
| Baseline LP method (fluoroscopy vs. non-fluoroscopy) | 0.38 (0.16, 0.93) | 0.0338 |
| Occurrence of LP-related AE within 7 days of baseline LP | 0.28 (0.15, 0.52) | <.0001 |
| **Healthy Control** | | |
| Time (1-year increase) | 0.51 (0.45, 0.59) | <.0001 |
| Sex (male vs. female) | 2.42 (1.07, 5.47) | 0.0331 |
| Site (US vs. non-US) | 3.42 (1.32, 8.85) | 0.0112 |
| **For participants with successful baseline LP only** (separate model) |  |  |
| Baseline LP method (fluoroscopy vs. non-fluoroscopy) | 0.02 (0.002, 0.19) | 0.0006 |

Report generated on data submitted as of: 17JUN2024.

The odds ratio (OR) and confidence interval (CI) estimates are obtained from generalized linear mixed models using LP success at each annual visit as the outcome.

*Due to the inclusion of interaction effects, the time estimate is calculated for a participant of average age (64.9 years) based at a US site.

**Supplementary Table 5. Reasons CSF not collected by cohort and visit**

| **Cohort / Visit** | **Participant declined** | **Participant unwell** | **Site issues** | **History of difficult LP** | **Spinal issues** | **Medical contraindications** | **Other** | **No reason provided** |
| --- | --- | --- | --- | --- | --- | --- | --- | --- |
| **Parkinson's Disease** | | | | | | | | |
| Baseline (N = 44) | 20 (59%) | 4 (12%) | 2 (6%) | 1 (3%) | 1 (3%) | 2 (6%) | 4 (12%) | 10 |
| Year 1 (N = 190) | 79 (59%) | 3 (2%) | 4 (3%) | 16 (12%) | 7 (5%) | 11 (8%) | 14 (10%) | 56 |
| Year 2 (N = 170) | 50 (49%) | 2 (2%) | 5 (5%) | 17 (17%) | 3 (3%) | 9 (9%) | 16 (16%) | 68 |
| Year 3 (N = 159) | 67 (54%) | 2 (2%) | 6 (5%) | 31 (25%) | 5 (4%) | 7 (6%) | 6 (5%) | 35 |
| Year 4 (N = 169) | 81 (53%) | 3 (2%) | 4 (3%) | 30 (19%) | 12 (8%) | 13 (8%) | 11 (7%) | 15 |
| Year 5 (N = 182) | 91 (51%) | 5 (3%) | 5 (3%) | 33 (19%) | 12 (7%) | 16 (9%) | 16 (9%) | 4 |
| Year 7 (N = 185) | 91 (50%) | 3 (2%) | 5 (3%) | 32 (17%) | 14 (8%) | 19 (10%) | 19 (10%) | 2 |
| Year 9 (N = 86) | 45 (52%) | 5 (6%) | 5 (6%) | 15 (17%) | 4 (5%) | 8 (9%) | 4 (5%) | 0 |
| Year 11 (N = 88) | 39 (44%) | 7 (8%) | 0 | 7 (8%) | 7 (8%) | 7 (8%) | 21 (24%) | 0 |
| Year 13 (N = 12) | 5 (NA) | 1 (NA) | 0 | 2 (NA) | 1 (NA) | 2 (NA) | 1 (NA) | 0 |
| **Prodromal** | | | | | | | | |
| Baseline (N = 42) | 7 (25%) | 2 (7%) | 2 (7%) | 0 | 0 | 5 (18%) | 12 (43%) | 14 |
| Year 1 (N = 95) | 37 (45%) | 2 (2%) | 3 (4%) | 15 (18%) | 4 (5%) | 10 (12%) | 11 (13%) | 13 |
| Year 2 (N = 93) | 37 (45%) | 0 | 1 (1%) | 23 (28%) | 5 (6%) | 5 (6%) | 12 (14%) | 10 |
| Year 3 (N = 84) | 39 (49%) | 0 | 4 (5%) | 20 (25%) | 4 (5%) | 6 (8%) | 7 (9%) | 4 |
| Year 4 (N = 92) | 47 (54%) | 0 | 0 | 21 (24%) | 3 (3%) | 5 (6%) | 11 (13%) | 5 |
| Year 5 (N = 107) | 55 (53%) | 1 (1%) | 2 (2%) | 22 (21%) | 4 (4%) | 7 (7%) | 13 (13%) | 3 |
| Year 7 (N = 54) | 26 (48%) | 1 (2%) | 0 | 5 (9%) | 2 (4%) | 2 (4%) | 18 (33%) | 0 |
| Year 9 (N = 20) | 8 (40%) | 1 (5%) | 0 | 4 (20%) | 0 | 1 (5%) | 6 (30%) | 0 |
| **Healthy Control** | | | | | | | | |
| Baseline (N = 2) | 0 | 0 | 0 | 0 | 0 | 0 | 2 (NA) | 0 |
| Year 1 (N = 25) | 2 (NA) | 0 | 0 | 1 (NA) | 0 | 1 (NA) | 3 (NA) | 18 |
| Year 2 (N = 36) | 5 (NA) | 0 | 0 | 1 (NA) | 1 (NA) | 1 (NA) | 4 (NA) | 24 |
| Year 3 (N = 32) | 5 (NA) | 0 | 0 | 2 (NA) | 2 (NA) | 1 (NA) | 0 | 22 |
| Year 4 (N = 43) | 20 (54%) | 0 | 0 | 2 (5%) | 8 (22%) | 3 (8%) | 4 (11%) | 6 |
| Year 5 (N = 41) | 22 (55%) | 0 | 1 (3%) | 6 (15%) | 5 (13%) | 4 (10%) | 2 (5%) | 1 |
| Year 7 (N = 50) | 27 (57%) | 0 | 0 | 6 (13%) | 6 (13%) | 2 (4%) | 6 (13%) | 3 |
| Year 9 (N = 19) | 4 (NA) | 2 (NA) | 1 (NA) | 8 (NA) | 2 (NA) | 2 (NA) | 0 | 0 |
| Year 11 (N = 30) | 14 (47%) | 0 | 1 (3%) | 7 (23%) | 1 (3%) | 0 | 7 (23%) | 0 |
| Year 13 (N = 12) | 6 (NA) | 0 | 0 | 4 (NA) | 0 | 1 (NA) | 1 (NA) | 0 |

Report generated on data submitted as of: 17JUN2024.

Percentages are not reported for groups with less than 20 participants
