# Supplementary figures and images for: Safety and Feasibility of Serial Lumbar Punctures: Long-term Results from the Parkinson’s Progression Markers Initiative
Source: Clin Park Relat Disord. 2025 Aug 13;13:100385. doi: 10.1016/j.prdoa.2025.100385 (PMC12391599; doi:10.1016/j.prdoa.2025.100385)

**Supplemental Figure 1: Longitudinal CSF Compliance by Cohort**


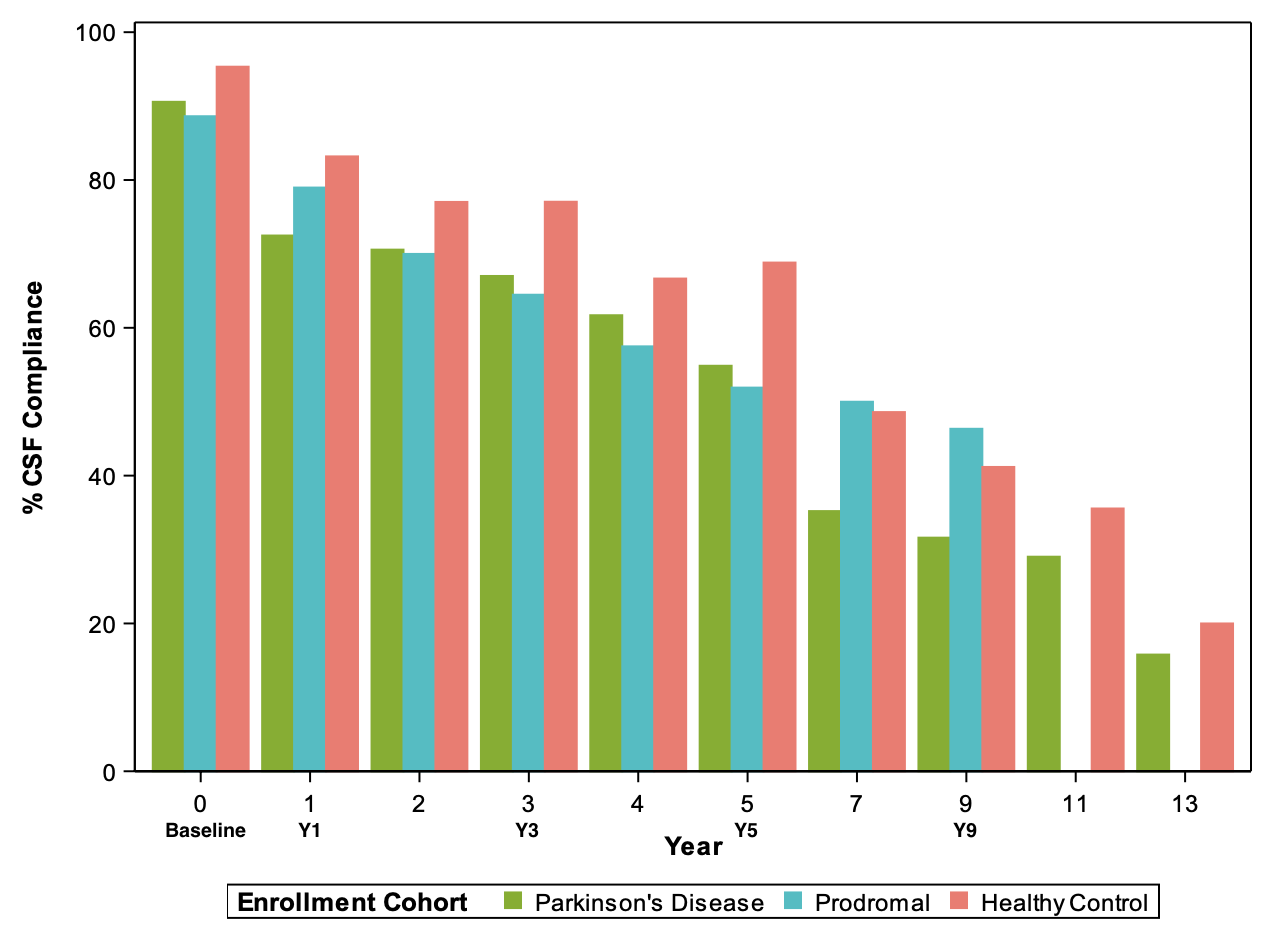

Supplement: Supplementary Data 3 [file mmc3.docx]
